# Tolerability and Efficacy of a Pediatric Granule Formulation of Artesunate-Mefloquine in Young Children from Cameroon with Uncomplicated Falciparum Malaria

**DOI:** 10.4269/ajtmh.2010.09-0704

**Published:** 2010-06

**Authors:** Félix Tietche, David Chelo, Njiki Kinkela Mina Ntoto, Florence Minjiwa Djoukoue, Christoph Hatz, Sarabel Frey, Adrian Frentzel, Sonja Trapp, Roland Zielonka, Edgar A. Mueller

**Affiliations:** Faculty of Medicine and Biomedical Sciences, University of Yaoundé 1, Yaoundé, Cameroon; Centre Mère et Enfant, Fondation Chantal Biya, Yaoundé, Cameroon; Swiss Tropical Institute, Basel, Switzerland; University Children's Hospital Basel, Switzerland; Mepha Ltd, Aesch, Switzerland; Institute for Clinical Pharmacology, Public Health Research Association Saxony, Technical University, Dresden, Germany

## Abstract

A fixed-dose pediatric formulation of artesunate and mefloquine (Artequin Pediatric) has been developed. In this open, non-comparative study in Cameroonian children with uncomplicated falciparum malaria, the safety and efficacy of this formulation was tested, with a particular emphasis on the risk of neuropsychiatric adverse events (AEs). In total, 220 subjects, weighing between 10 and 20 kg, were enrolled; 213 qualified for analysis. Artesunate-mefloquine was given once daily for 3 days. Overall, 13.1% of patients reported mild to moderate neuropsychiatric AEs (elicited through a structured questionnaire or reported spontaneously) out of which 3.8% (mainly insomnia) were considered drug-related. Other drug-related AEs were infrequent (< 3%). Polymerase chain reaction-corrected cure rate (adequate clinical and parasitological response) determined by survival analysis at 28 and 63 days was 96.6%. New infections were observed in 11.2% of evaluable patients at 63 days. The new formulation was well tolerated and efficacious in the population investigated.

## Introduction

*Plasmodium falciparum* malaria is a leading cause of mortality in tropical countries, with most of the deaths occurring in children from sub-Saharan Africa.^1^–^3^ As resistance to available antimalarial drugs worsens, morbidity and mortality rises as a direct consequence.^4^ In 2006 the World Health Organization (WHO) recommended the use of artemisinin-based combination therapy (ACT) as the first-line treatment of uncomplicated *P. falciparum* malaria in all endemic areas because of its efficacy and potential to reduce the spread of drug resistance.^5^ The ACTs are currently the most preferred treatments for uncomplicated falciparum malaria, and many sub-Saharan African countries have already implemented ACTs as first line treatments.^4^,^6^–^9^

Artesunate-mefloquine is an ACT, which has been widely used in Asia; but also in Africa this combination appears to be highly efficacious in patients with uncomplicated *P. falciparum* malaria.^4^,^10^ The recommended treatment is 4 mg/kg body weight of artesunate given once a day for 3 days and 25 mg/kg of mefloquine split over 2 or 3 days.^5^ Recently, a novel fixed-dose pediatric co-formulation of artesunate and mefloquine (Artequin Pediatric, Mepha, Switzerland) was developed. This stickpack formulation (i.e., a thin, tube-shaped foil pack) contains mango-flavored artesunate (50 mg) and mefloquine (125 mg) in separate granules, which may be administered directly into the mouth of the patient. It is a further development of the co-blister tablet preparation (Artequin), which proved to be highly efficacious and well tolerated in patients from Southeast Asia^11^ and Africa^12^–^16^ when given for 3 days. In a pharmacokinetic study in pediatric patients, the fixed-dose pediatric formulation and the standard co-blister of artesunate-mefloquine delivered comparable systemic exposure to the active moieties dihydroartemisinin and mefloquine.^17^

Because of insufficient safety and tolerability data for artesunate-mefloquine, there has been concern regarding the use of this combination in African children, which restricted its deployment in this setting.^5^,^10^ One reason for this data gap is the lack of an appropriate pediatric formulation.^18^ Another reason is the concern that artesunate-mefloquine may be poorly tolerated by young children, although it has been previously suggested that children tolerate ACTs as well as or even better than adults.^4^ In some malaria treatment trials, mefloquine monotherapy caused a higher rate of certain gastrointestinal (GI) and central nervous system (CNS) adverse events (AEs) compared with chloroquine (dizziness), halofantrine (nausea, vomiting, fatigue, dizziness) and artemether-lumefantrine (nausea, vomiting, dizziness, insomnia).^19^ In particular, the potential neuropsychiatric effects of mefloquine attracted attention in the past.^20^–^22^ Likewise, when mefloquine was combined with an artemisinin derivative to treat malaria, dizziness was commonly reported by adults from Southeast Asia; however, it was generally not considered clinically relevant or caused by the underlying malaria disease.^23^,^24^ In African adults, dizziness was less likely to occur when mefloquine was combined with artesunate compared with mefloquine monotherapy.^25^

Concerns have also been raised by preclinical observations of neurotoxicity with artemisinin derivatives. In animal studies, intramuscular oil-based artemether and arteether have been associated with neuronal damage, particularly in areas of the brainstem involved in hearing and gait control^7^; however, the findings have never been confirmed in humans.

Against this background, the safety and efficacy of the new granule formulation of artesunate-mefloquine was investigated in young African children with uncomplicated *P. falciparum* malaria. The primary aim was to assess the neuropsychiatric safety of the new pediatric formulation. Major secondary outcomes included Day-28 and Day-63 polymerase chain reaction (PCR)-corrected cure rates.

## Methods

### Study site.

The study was performed between December 2007 and March 2009 in Yaoundé, Cameroon, where malaria transmission is intense and perennial.^26^ Patients with microscopically confirmed acute uncomplicated *P. falciparum* malaria were recruited from the peri-urban area of Yaoundé.

### Study population.

Male or female children presenting with signs and symptoms of malaria were screened for study eligibility. Inclusion criteria were body weight between 10 and 20 kg, fever (temperature ≥ 37.5°C axillary or ≥ 38°C orally, rectally or tympanically) or history of fever in the preceding 24 hours, *P. falciparum* malaria with a density between 2,000/μL (amended to 1,000/μL) and 250,000/μL blood, ability to take drugs by mouth and to comply with the study protocol, and provision of written informed consent by the parent or guardian. Exclusion criteria included presence of severe and complicated malaria (e.g., cerebral malaria) as defined by WHO,^27^ history of serious side effects related to artesunate-mefloquine or similar drugs, use of antimalarial drugs or any other agent with antimalarial activity within the previous week, ingestion of mefloquine within the previous 30 days, serious underlying disease, presence of severe vomiting or diarrhea, history of epilepsy, convulsion or splenectomy, and need for parenteral treatment.

Before enrollment, written informed consent was obtained from the parents or legal guardians of the child. The trial protocol was approved by the National Ethics Committee, Yaoundé, Cameroon and the Ethics Committee of the Technical University of Dresden, Medical Faculty, Germany. This study is registered with ClinicalTrials.gov as NCT00978172.

### Study design and procedures.

This was an open-label, single-center, single-arm study. Patients were enrolled consecutively and admitted to the health facility for the first day of a 3-day treatment phase, and then followed up until Day 63 after treatment. A follow-up period of 63 days complied with the WHO guideline from 2003 recommending this observation period for patients treated with artesunate-mefloquine to avoid an underestimation of recrudescence rates.^28^ Those patients recruited between December 2007 and June 2008 (*N* = 50) were included in a prospectively planned interim analysis. The data from these patients were reviewed by an independent malaria expert and a neuropediatrician to confirm or reassess the initially planned sample size of 600 patients, and to look for insufficient efficacy and potential safety issues.

The investigational therapy (Artequin Pediatric) consisted of artesunate 50 mg/day (2.5–5 mg/kg/day) and mefloquine 125 mg/day (6.25–12.5 mg/kg/day) given in equal oral doses simultaneously in a fixed-dose formulation (stickpack with a mixture of artesunate and mefloquine granules) once daily for 3 consecutive days. The entire content of one stickpack was directly administered on the child's tongue. In case of administration difficulties, the content was to be put on a spoon and administered with a small amount of liquid. The mouth was thereafter to be rinsed with some liquid, e.g., with milk or water, and remaining granules swallowed. Patients have been encouraged to resume normal food intake as soon as food could be tolerated. The first dose was administered under supervised conditions at the health facility. The second and third doses were given at home by the parent or guardian. The two stickpacks for administration at home had to be returned to the study site (used or not) to check for treatment compliance. Patients who vomited a dose within 30 minutes after intake received a full replacement dose. No more than one dose was to be replaced per day. The study medication was provided by Mepha Ltd., Aesch, Switzerland in Good Manufacturing Practice quality.

Parents or guardians were asked to bring their children back to the health center on Days 4, 7, 28, and 63 or on any other day if the child did not feel well. Rescue therapy according to local practice was administered to patients with early or late treatment failures,^28^ new falciparum infection, and in case of vomiting the study replacement dose. In case of premature discontinuation of study medication, information on the status of the patient was collected according to the scheduled study visits. Vital signs and hematology parameters were assessed at baseline and each follow-up visit. At baseline and at every follow-up visit, potential AEs were assessed for severity and association with study medication.

Neuropsychiatric examinations took place at baseline and on Days 7, 28, and 63. These examinations included standardized questions (in local language) to the parent or guardian concerning their observations, questions to the children according to their age and development status, doctor's observations, and standardized neurological assessments.^29^–^32^ Apart from neurological abnormalities, the neuropsychiatric examination included an anamnesis/questionnaire looking for sleeping disorders, neurocognitive and behavioral disturbances, speech disturbances, and eating disturbances. All neuropsychiatric symptoms that occurred newly or worsened from baseline were recorded as AEs or serious adverse events (SAEs), independent whether elicited through the structured questionnaire or spontaneously reported by the patient or parent/guardian.

Giemsa-stained thick and thin blood films were examined before initiation of treatment and at every follow-up visit (scheduled or not). Blood films were considered negative if no parasites were seen in 100 oil-immersion fields in a thick blood film. The thin film was used for species determination. Two qualified microscopists independently read the slides. In case of discrepant results, slides were additionally read by a parasitologist.

To distinguish between recrudescence and new infection, blood samples for PCR analysis were collected from every patient at baseline and on Days 4, 7, 28, and 63. Additional PCR samples were taken in case the patient spontaneously returned to the health unit for signs and symptoms of malaria. For PCR analysis, a common technical protocol was used.^33^

### Discussion of study design.

An open-label, non-comparative design was adopted as done previously with ACTs in this vulnerable population.^34^,^35^ At the time of the study, no suitable pediatric formulation of an ACT was available (i.e., approved by regulatory authorities), which could have acted as comparator. In addition, it was expected that clinical judgment can be applied to assess the clinical relevance of the rate of occurrence of neuropsychiatric events, the primary aim of the trial. Neuropsychiatric AEs were assessed in a way appropriate for children, according to their age and developmental status with a standardized questionnaire and examination. The study lasted for 63 days, which represents three half-lives of mefloquine.^36^

### Study endpoints.

The primary objective of the trial was to assess the neuropsychiatric safety of the pediatric granule formulation of artesunate-mefloquine based on the incidence of neuropsychiatric AEs. Efficacy outcomes represented secondary study endpoints. Therapeutic outcomes were classified according to the current WHO protocol (WHO 2003) and included the proportion of patients who showed an adequate clinical and parasitological response (ACPR) at 28 and 63 days (PCR corrected and uncorrected). The ACPR was defined as absence of parasitemia on Day 28 (or Day 63) irrespective of axillary temperature, without any previous occurrence of early treatment failure, late clinical failure, or late parasitological failure.

### Statistical analysis.

A conservative 40% rate of neuropsychiatric AEs was assumed for the study treatment based on AE frequencies (mainly dizziness) reported for mefloquine when the drug was combined with an artemisinin derivative in adults from Southeast Asia.^23^,^24^ With a study size of 600 patients, the two-sided 95% confidence interval (CI) was estimated to be approximately 36–44% when the observed rate would actually be 40%. An interim analysis was prospectively planned to confirm or reassess the initially planned sample size. On the basis of this interim analysis (*N* = 50), the study size was reduced from 600 to 200 patients because of the low incidence of neuropsychiatric AEs overall and the virtual absence of drug-related neuropsychiatric AEs.

This was a single-arm study; hence, no pre-defined statistical hypothesis was tested. Consequently, no adjustment for multiple testing was made and two-sided 95% CIs were calculated for the primary safety variable (i.e., neuropsychiatric AEs). Day-28 and Day-63 ACPR rates were analyzed by the Kaplan-Meier method.^28^ Patients who discontinued study participation without fulfilling the criteria of early treatment failure, late clinical failure, or late parasitological failure (e.g., losses to follow-up), or to whom another antimalarial drug was administered without fulfilling these criteria were considered censored at the time of withdrawal/start of rescue antimalarial medication. For analysis of the PCR-corrected rates, re-infections were additionally classified as censored observations in the Kaplan-Meier analysis.

Data were analyzed on the basis of different populations. The analysis of the primary safety endpoint was based on a modified safety population, defined as all patients who took at least one dose of study medication and had a post-baseline neuropsychiatric assessment on Day 7 or who reported a drug-related neuropsychiatric AE at any time before a new malaria episode occurred. The safety population included all patients who received at least one dose of study drug and had at least one post-baseline assessment, regardless of whether scheduled or unscheduled. A modified intention-to-treat (ITT) population was used for analyzing ACPR rates, and comprised all patients who had positive parasitemia at baseline and took at least one dose of study medication. Patients who discontinued the study before Day 4 without previous occurrence of early treatment failure or who had a mixed strain infection (as assessed by PCR) were excluded.

## Results

In total, 220 patients with uncomplicated falciparum malaria were enrolled; 83.6% of patients completed the 63-day study (Table 1). Main reasons for patients discontinuing the study prematurely were loss to follow-up and the presence of a new malaria episode. The 3-day treatment period was completed by 96.8% of patients (*N* = 213), and 38.9% of the patients (14 of 36) who prematurely discontinued the study participation did so at or after Day 28. Only two patients discontinued the study because of an AE (severe malaria and convulsions), both occurred during the 3-day treatment period but were considered unrelated to the study drug. Most patients (197 of 220, 89.5%) adhered to the visit schedule throughout the study, and only a low proportion of patients (< 1%) returned unused stickpacks to the study site. The mean (range) actual antimalarial doses in mg/kg/day were 3.5 (2.5–5) for artesunate and 8.8 (6.25–12.5) for mefloquine.

For both, the modified safety population and the modified ITT population, 213 (96.8%) participants qualified for inclusion. The main reason for exclusion was missing follow-up data for any visit (2.3%). In total, 215 (97.7%) patients were included in the safety population. Baseline demographics and background characteristics are shown in Table 2. There was a balanced gender distribution. Approximately half of the patients were 1.5–3.5 years of age with an overall mean of 3.2 years (median: 3.1 years). The age ranged between 8 months and 8 years; five patients were younger than 1 year. Mean parasite density was 36,476/µL (median: 20,040/µL). Most common malaria symptoms at baseline were fatigue (84.5% of patients), anorexia (70.4%), fever (60.6%), and anemia (46.5%). Vomiting at baseline was observed in 24.9% of patients. The study population recruited represented the target population in countries where malaria is endemic.

### Tolerability and safety.

Artesunate-mefloquine was well tolerated by the study participants. Only two children vomited during the 3-day treatment period. One patient vomited the study drug and the replacement dose on Day 1, another patient vomited the Day-2 study medication.

In total, 28 patients reported 50 neuropsychiatric AEs over the study course (Table 3), which represented 13.0% (95% CI: 9.0 to 18.0%) of the safety population and 13.1% (95% CI: 8.9 to 18.4%) of the modified safety population. All these AEs were newly emerging. The most frequent neuropsychiatric AE was sleeplessness/insomnia, which was reported for 7.0% of patients, followed by altered eating behavior (4.7%) and headache (3.7%); 35 of 50 neuropsychiatric AEs started ≥ Day 10, 30 of 50 ≥ Day 20, and 25 of 50 ≥ Day 30. In the majority of cases, these AEs lasted for 3–6 days. There was no marked difference between the different age groups in terms of incidence rates (Table 3). It appeared that very young children were less frequently affected but the number of patients with assessments was low (*N* = 27). Almost all of the neuropsychiatric AEs were either mild or moderate in severity. One child suffered from severe convulsion, which was considered unrelated to study medication and caused by the underlying malaria disease. The event resolved within 2 days on treatment with intravenous quinine.

The incidences of drug-related neuropsychiatric AEs (primary safety variable) are shown in Table 3. Out of the 50 neuropsychiatric AEs, 11 were suspected to be study drug-related and occurred in 8 patients. This represented 3.7% (95% CI: 2.0 to 7.0%) of the safety population and 3.8% (95% CI: 1.6 to 7.3%) of the modified safety population. The most frequent drug-related neuropsychiatric AE was sleeplessness/insomnia, which was reported in 4 (1.9%) patients. In addition, single episodes of dizziness, headache, vertigo, nightmares, hyperactivity, aggressive behavior, and altered eating behavior were reported. Most of these drug-related AEs started within the first 10 days of the study and usually lasted for 4 to 7 days; all of them showed a spontaneous resolution. There was no difference between the different age groups in terms of incidence rates. All drug-related neuropsychiatric AEs were of mild to moderate severity.

A total of 169 AEs other than neuropsychiatric were reported in 112 patients (52.1%). The most frequently affected system organ classes were infections and infestations (27.0%) and blood and lymphatic system disorders (20.5%). The most frequent AEs were anemia (20.5%), bronchitis (7.0%), and pyrexia (6.5%); vomiting was reported by 4 (1.9%) patients. Most of these AEs were either mild or moderate in severity. The AEs were not unexpected in children suffering from acute uncomplicated malaria and were largely pre-existing at baseline. Eleven [reported in 10 (4.7%) patients] of the 169 non-CNS AEs were classified as related to the study drug. All of the AEs suspected to be drug-related were mild or moderate in severity. The most frequent drug-related AEs were anemia (6 patients, 2.8%), diarrhea (2 patients, 0.9%) and vomiting (2 patients, 0.9%); they all had an onset within the first week of the study and lasted for 1–3 days, except anemia, which lasted 3–4 weeks.

Overall, six SAEs occurred in 5 (2.3%) patients. One patient suffered from two episodes of anemia requiring hospitalization. Both episodes occurred in the context of a malaria infection. Two patients were hospitalized for severe malaria in the context of re-infections, one child was hospitalized because of severe asthenia related to the baseline malaria infection, and one patient suffered from convulsions during the night after baseline assessment and was hospitalized. All six SAEs were classified as not related to the study drug and resolved by the time of the last follow-up visit. No patient died.

Changes of vital signs and hematology parameters were in accordance with malaria recovery.

### Efficacy.

Cure rates were high (Table 4). The PCR-corrected ACPR rates were 96.6% for both Day 28 and Day 63 (Kaplan-Meier analysis). Simple proportions of PCR-corrected 28-day and 63-day ACPR rates for evaluable patients were 96.5% (194 of 201) and 96.0% (168 of 175), respectively. In one patient, parasitemia was present at Day 15 but no PCR result was available. This patient was classified as having a recrudescence. Three other patients revealed a PCR-confirmed recrudescence on Days 26, 11, and 18, respectively. All four recrudescences were associated with typical signs and symptoms of malaria, and were therefore classified as late clinical failures. Three patients (1.4%) were classified as early treatment failures. One patient vomited the study drug and the replacement dose at Day 1. Another presented with convulsions during the night after baseline, and was treated with intravenous quinine. Finally, one child developed severe malaria and was also treated with intravenous quinine. In a conservative approach, these three patients with early treatment failure were classified as recrudescences. No patient developed late parasitological failure.

In the non-PCR corrected analysis, the ACPR rates were 96.1% for Day 28 and 85.4% for Day 63 (Kaplan-Meier analysis). There were 22 patients with a PCR-confirmed new *P. falciparum* infection up to Day 63 (11.2% of 197 evaluable patients). Mean time to re-infection was 52 ± 11 days (median: 56 days); there was only one re-infection by Day 28. The most frequently used rescue medication for treatment failure or new infection was artemether/lumefantrine (3.3% of patients).

## Discussion

The assessment of the safety and efficacy of a new pediatric granule formulation of artesunate-mefloquine in the treatment of uncomplicated falciparum malaria in African children weighing 10–20 kg was the main purpose of this study. The new formulation was well tolerated and highly efficacious in the treatment of falciparum malaria in young Cameroonian children. Very few patients (3.8%) reported drug-related neuropsychiatric AEs out of which sleeplessness/insomnia of mild to moderate severity occurred most frequently. All non-drug-related neuropsychiatric events were likely to be related to the underlying malaria disease. Only 4.7% of patients reported non-CNS AEs suspected to be drug-related, all of mild or moderate severity; mainly anemia and gastrointestinal disorders, which also may have been attributable to the underlying malaria infection. Non-drug-related, non-CNS AEs were observed but all of them were expected in children suffering from acute uncomplicated malaria and were largely pre-existing at baseline. No new safety signal was detected.

Data on neuropsychiatric effects of mefloquine in young children are limited. One study performed in Southeast Asia found no evidence for such effects in children less than 5 years of age (weighing > 5 kg), when mefloquine, either alone or combined with an artemisinin derivative, was administered to treat falciparum or vivax malaria.^37^ In a recent study, neither artesunate nor mefloquine resulted in significant impairment of behavior or motor function in Karen children, aged between 3 months and 5 years, when compared with non-febrile controls.^38^ Our study in young African children confirmed these findings. We observed a low incidence of neuropsychiatric AEs; none represented a safety concern. In addition, the study corroborated the absence of clinically relevant neurotoxic effects caused by oral artemisinin derivatives in general and artesunate in particular.^39^,^40^ However, the number of African children treated with artesunate-mefloquine is still relatively small, and the occurrence of rare and potentially severe AEs cannot be completely ruled out.

Vomiting of antimalarial drugs is an important consideration in treatment. Mefloquine may cause two types of vomiting: early vomiting (< 1 hour after intake) related to gastric intolerability and late vomiting (> 1 hour and up to Day 7) probably related to central effects and co-existence of disease-related symptoms.^41^ In this study, vomiting occurred in 4 (1.9%) patients, with only two children vomiting early. This low proportion is in agreement with previous studies in adolescents and adults from Africa using the artesunate-mefloquine co-blister preparation.^12^,^13^,^15^,^16^ The other types of reported AEs also were comparable to the ones that have been described in African patients using the co-blister,^12^–^16^ and were most commonly symptoms of malaria. Likewise, the pattern of changes seen in clinical laboratory parameters was consistent with acute malaria and its resolution.

As expected, the efficacy of the new artesunate-mefloquine pediatric formulation was excellent, with PCR-corrected ACPR rates of 96.6% for both Day 28 and Day 63, thereby indicating the absence of recrudescence after Day 28. In adolescents and adults, high Day-28 cure rates have been previously reported for the artesunate-mefloquine co-blister preparation.^12^–^16^ In one study, a Day-42 ACPR rate of 100% was shown in a small subgroup of patients.^14^ Short observation periods may yield an underestimation of recrudescence rates. Hence, we chose a long follow-up period of 63 days, which deserves special attention. The high PCR-adjusted cure rate over 63 days suggests a sustained efficacy with artesunate-mefloquine in young African children, possibly caused by the long half-life of mefloquine; although undetected, transient recrudescences between Days 28 and 63 could have occurred. The results were in accordance with those of a large study previously published: van Vugt and colleagues^42^ reported a Day-63 PCR-adjusted cure rate of 94% in patients from Southeast Asia. In a more recent study in the same population, a lower Day-63 PCR-corrected success rate was observed with artesunate-mefloquine (approximately 90%), which may reflect a decreased sensitivity to mefloquine in Southeast Asia or chance fluctuations.^43^

It has been suggested that ACT partner drugs with longer prophylactic times could result in a larger impact in higher-transmission settings, although their long-term benefit must be evaluated in relation to the risk of parasite resistance.^44^ In this context, the re-infection rates observed in our study appear of interest. Only 11% of evaluable patients showed a re-infection at 63 days, which is remarkably low in high malaria transmission areas. In a recent large, randomized study in East African children (Tanzania), the effectiveness of amodiaquine (*N* = 270), amodiaquine+sulfadoxine-pyrimethamine (*N* = 507), amodiaquine+artesunate (*N* = 515), and artemether-lumefantrine (*N* = 519) was compared.^45^ Re-infection rates at Day 28 were 28%, 27%, 31%, and 18%, respectively, and thus markedly higher than in this study, which lasted 5 weeks longer. In West Africa, an unadjusted 42-day risk of treatment failure of 12.2% for dihydroartemisinin-piperaquine was reported in children < 5 years of age from Burkina Faso.^46^ Our results confirm the expected prevention of re-infection by mefloquine over a prolonged period of time, comparable to that of dihydroartemisinin-piperaquine.

The administration of study medication was largely done under non-supervised conditions (i.e., the administration of two of the three drugs took place at home). This is close to normal outpatient practice. The limitations of the study included the absence of an active control arm, which makes it difficult to estimate relative safety and efficacy in comparison with other treatments. In addition, it was an open-label study, which is associated with the unavoidable risk of bias, particularly with regard to AE reporting. However, the use of a standardized questionnaire adapted according to age and developmental status, and the conduct of standardized neurological examinations lowered the risk of bias. Moreover, the fact that neuropsychiatric AEs were elicited (not only spontaneously reported) carried the risk for over-reporting of such AEs; whereas certain signs and symptoms, which are difficult to assess in very young children (e.g., dizziness), may have been under-reported in this age group.

In conclusion, over a 2-month observation period, the new fixed-dose pediatric granule formulation of artesunate-mefloquine administered once daily for 3 consecutive days was well tolerated and highly efficacious for the treatment of acute uncomplicated *P. falciparum* malaria in young Cameroonian children weighing between 10 and 20 kg. A small proportion of patients reported drug-related neuropsychiatric AEs of which sleeplessness/insomnia occurred most frequently. Despite earlier restrictions, there seems to be no reason to withhold artesunate-mefloquine from this population.

## Figures and Tables

**Table 1:** Patient disposition (enrolled patients)

	*N* = 220 n (%)
Completed 63-day study	184 (83.6)
Discontinued study before Day 63	36 (16.4)
Adverse event	2 (0.9)
Lost to follow-up	16 (7.3)
Moved from study area	1 (0.5)
New episode of malaria	12 (5.5)
Subject withdrew consent	3 (1.4)
Protocol violation	2 (0.9)

**Table 2:** Baseline demographics and background characteristics of patients*

	*N* = 213
Sex n (%)	
Male	104 (48.8)
Female	109 (51.2)
Race n (%)	
African	213 (100)
Mean age (yr)	3.2
SD	1.5
Range	0.7–7.9
Mean weight (kg)	14.2
SD	2.8
Range	10–20
Age categories n (%)	
< 1.5 years	27 (12.7)
≥ 1.5 to < 3.5 years	104 (48.8)
≥ 3.5 to < 5 years	51 (23.9)
≥ 5 years	31 (14.6)
Mean height (cm)	*N* = 212 94.8
SD	11.5
Range	72–123
Mean parasite density, asexual forms (/µL)	36,476
SD	43,593
Range	1,000–248,800

* For modified safety population (i.e., all patients who took at least one dose of study medication and had a post-baseline neuropsychiatric assessment on Day 7 or who reported a drug-related neuropsychiatric adverse event at any time before a new malaria episode occurred).

**Table 3:** Patients with at least one neuropsychiatric adverse event*

	All neuropsychiatric AEs n/*N* (%) [95% CI]	Drug-related neuropsychiatric AEs n/*N* (%) [95% CI]
Total	28/213 (13.1) [8.9 to 18.4]	8/213 (3.8) [1.6 to 7.3]
< 1.5 years	2/27 (7.4) [0.9 to 24.3]	1/27 (3.7) [0.1 to 19.0]
≥ 1.5 to < 3.5 years	15/104 (14.4) [8.3 to 22.7]	4/104 (3.9) [1.1 to 9.6]
≥ 3.5 to < 5 years	6/51 (11.8) [4.4 to 23.9]	2/51 (3.9) [0.5 to 13.5]
≥ 5 years	5/31 (16.1) [5.5 to 33.7]	1/31 (3.2) [0.1 to 16.7]

* For modified safety population (i.e., all patients who took at least one dose of study medication and had a post-baseline neuropsychiatric assessment on Day 7 or who reported a drug-related neuropsychiatric AE at any time before a new malaria episode occurred).

AE = adverse event; CI = confidence interval.

**Table 4:** Proportion of patients with ACPR*

	Kaplan Meier estimate	95% CI
ACPR-PCR corrected (%)		
Day 28	96.6	93.0 to 98.4
Day 63	96.6	93.0 to 98.4
ACPR-uncorrected (%)		
Day 28	96.1	92.4 to 98.0
Day 63	85.4	79.7 to 89.7

* For modified intention-to-treat population (i.e., all patients who had positive parasitemia at baseline and took at least one dose of study medication; patients who discontinued the study before Day 4 without previous occurrence of early treatment failure or who had a mixed strain infection were excluded).

ACPR = adequate clinical and parasitological response; CI = confidence interval; PCR corrected = polymerase chain reaction method was used to exclude new *P. falciparum* infections from this analysis.
